# Proficiency testing for bacterial whole genome sequencing: an end-user survey of current capabilities, requirements and priorities

**DOI:** 10.1186/s12879-015-0902-3

**Published:** 2015-04-03

**Authors:** Jacob Moran-Gilad, Vitali Sintchenko, Susanne Karlsmose Pedersen, William J Wolfgang, James Pettengill, Errol Strain, Rene S Hendriksen, on behalf of the Global Microbial Identifier initiative’s Working Group 4 (GMI-WG4)

**Affiliations:** Public Health Services, Ministry of Health, Jerusalem, Israel; Surveillance and Pathogenomics Israeli Centre of Excellence (SPICE), National Institute for Biotechnology in the Negev, Ben-Gurion University, Beer-Sheva, Israel; Sydney Medical School and Marie Bashir Institute for Infectious Diseases and Biosecurity, University of Sydney, Sydney, Australia; Centre for Infectious Diseases and Microbiology – Public Health, Institute of Clinical Pathology and Medical Research – Pathology West, Westmead Hospital, Sydney, Australia; Technical University of Denmark, National Food Institute, Research Group of Microbial Genomics and Antimicrobial Resistance, National Food Institute, Technical University of Denmark, Kemitorvet, Building 204, ground floor, DK - 2800 Kongens Lyngby, Denmark; Bacteriology Laboratory, Wadsworth Center, New York State Department of Health, Albany, New York USA; Biostatistics Branch, US Food and Drug Administration, College Park, Maryland USA

**Keywords:** Proficiency, Bioinformatics, Next-generation sequencing, Survey, End-users, Public health, Microbiology, Standards

## Abstract

The advent of next-generation sequencing (NGS) has revolutionised public health microbiology. Given the potential impact of NGS, it is paramount to ensure standardisation of ‘wet’ laboratory and bioinformatic protocols and promote comparability of methods employed by different laboratories and their outputs. Therefore, one of the ambitious goals of the Global Microbial Identifier (GMI) initiative (http://www.globalmicrobialidentifier.org/) has been to establish a mechanism for inter-laboratory NGS proficiency testing (PT). This report presents findings from the survey recently conducted by Working Group 4 among GMI members in order to ascertain NGS end-use requirements and attitudes towards NGS PT. The survey identified the high professional diversity of laboratories engaged in NGS-based public health projects and the wide range of capabilities within institutions, at a notable range of costs. The priority pathogens reported by respondents reflected the key drivers for NGS use (high burden disease and ‘high profile’ pathogens). The performance of and participation in PT was perceived as important by most respondents. The wide range of sequencing and bioinformatics practices reported by end-users highlights the importance of standardisation and harmonisation of NGS in public health and underpins the use of PT as a means to assuring quality. The findings of this survey will guide the design of the GMI PT program in relation to the spectrum of pathogens included, testing frequency and volume as well as technical requirements. The PT program for external quality assurance will evolve and inform the introduction of NGS into clinical and public health microbiology practice in the post-genomic era.

**Electronic supplementary material:**

The online version of this article (doi:10.1186/s12879-015-0902-3) contains supplementary material, which is available to authorized users.

## Background

The advent of next-generation sequencing (NGS) technologies has revolutionised molecular microbiology by making genome sequences of pathogens of clinical or public health importance, readily available [1]. NGS has many advantages over other existing molecular approaches, including throughput, quality, flexibility, scalability and thus may potentially replace a multitude of assays currently run simultaneously in a diagnostic microbiology laboratory [2,3].

Translation of NGS from research centres to public health and clinical laboratories has already begun. As the technology becomes less expensive and turnaround times shorten, expansion of NGS into diagnostic practice is expected to be rapid. The first significant role for NGS is likely to be in the communicable disease surveillance and outbreak investigations [4]. Recent studies have demonstrated that SNPs mined from whole genome sequence (WGS) data [5-7] as well as gene-by-gene (core genome multi-locus sequence typing (MLST) [8]) comparisons provided far greater resolution for outbreak detection and for microbial strain tracking for a wide range of bacterial pathogens than current gold standards such as pulsed-field gel electrophoresis (PFGE), spoligotyping, and variable number tandem repeat-based typing. Additionally, the growth of public databases harbouring reference genomes continues to enhance the utility of NGS in public health and in clinical practice [9,10]. Thus NGS technologies will undoubtedly improve molecular epidemiology studies, public health laboratory surveillance and communicable disease control in the future [11-13].

This paradigm shift in clinical diagnostics and surveillance of microorganisms as a result of the rapid development of inexpensive NGS technologies and continuing increase in computing power and data-transport capacity will impact microbiology in clinical laboratories, hospitals and other public health institutions. Ideally, it will also enable all countries to detect current and emerging infectious diseases in real-time and at low cost and share information in a standardised manner [14]. Thus, an initiative was started in September 2011 by several infectious disease control centres and other organisations with the first meeting convened in Brussels formulating the overall goal [15]; A global system to aggregate, share, mine and translate genomic data for microorganisms in real-time [14]. Since then, the initiative has grown and is today composed by over 150 experts from around 30 countries. Subsequently, the initiative was named; the Global Microbial Identifier (GMI) and a Steering Committee was established as well as five working groups.

Given the expectation for a growing reliance on NGS technologies in clinical and public health laboratories it is paramount to understand and assess the robustness of results from different methodologies in order to enhance standardisation of ‘wet’ laboratory and bioinformatics analyses and promote comparability [16]. Therefore, one of the goals of the GMI initiative is to establish a formal mechanism for inter-laboratory test performance to ensure harmonisation and standardisation in WGS and data analysis. In February 2013 at the GMI initiative’s 5^th^ meeting in Copenhagen Denmark, a visionary taskforce of scientists and other stakeholders met, sharing an aim of making novel genomic technologies and bioinformatics tools available for improved global patient diagnostics, surveillance and research, by developing data exchange and analysis tools for characterisation of all microbial organisms and microbial communities.

During this meeting, the GMI Working Group 4 (WG4) was established to coordinate the GMI sponsored proficiency testing (PT) exercises. By having multiple laboratories perform NGS on a set of well-characterised strains, the results produced by the different laboratories will be used to identify those steps in the process where QA/QC (quality assurance/quality control) measures need to be taken to increase the concordance among results and harmonise the interpretation of data. To ensure any PT exercise was aligned with the expectation of the GMI end-users, a survey was developed to identify the types of end-user, the priority test organisms and quality markers to be measured. This report outlines the results of this survey of GMI members (survey available as supplementary file) in relation to their current capabilities, requirements for and attitudes towards performance of PT.

## Methods

With the aim to ensure harmonisation and standardisation in WGS and data analysis, WG4 developed a survey using the online survey software (https://www.surveymonkey.com/) for the collection of relevant information from scientists based in institutes and organisations from different parts of the world (supporting information). It included questions within three main topics with responses allowing for: 1) identification of potential end users of a PT, 2) identification of target organisms to be sequenced during a PT, and 3) establishment of quality assessment procedures to be implemented in the PT. Differences in responses within an organisation were likely to be observed and therefore respondents were encouraged to submit data as individuals/research groups within institutions. The respondents were encouraged to submit information on their needs and capacity in relation to DNA preparation, sequencing, and analysis (e.g. variant detection and clustering) enabling the organisers to take this information into consideration when creating the PT and in the work towards standardised testing and quality assurance of these tests.

The questionnaire contained 35 items, provided in three sections, including information on end-users (personal and organisational information), characterisation of target organisms and quality assessment. Specifically, information was sought regarding the institutional profile of respondents, capability and capacity of performing NGS, institutional priorities for NGS, attitudes towards a PT for NGS, operational aspects of delivering a PT for NGS and finally a survey of current technical NGS and bioinformatics practices. The responses were collected as free text or single options from pre-defined drop-down lists. Some responses were measured on a 5-point Likert-type scale with anchors specific to the question (e.g., 1 = strongly agree; 2 = agree; 3 = unsure; 4 = disagree; 5 = strongly disagree). Pilot testing was done with WG4 members to determine the acceptability and clarity of the questionnaire. The questionnaire is available as an appendix to this report.

Invitations to participate in the survey were sent to members of the Global Microbial Identifier initiative worldwide (N = 155) with a link directing to the online survey. No monetary incentive was offered. The invitation included information that responses would be kept confidential and would be anonymised prior to inclusion in a published report. The survey was available online for a two month period during which electronic invitations and reminders were sent to those who had not responded. Respondents were invited to send any questions or feedback for the survey to the organisers. Data were collected by https://www.surveymonkey.com/ and responses were downloaded both as summaries and detailed Excel spreadsheets.

## Results

### Profile of respondents

In all, 47 responses were registered in the system. Following de-duplication, 45 responses were eligible for analysis, representing an overall survey response rate of 29%. The distribution of respondent’s country of origin was as following: United States (n = 14, 31.1%), United Kingdom (n = 7, 15.6%), Denmark (n = 4, 8.9%), Canada (n = 4, 8.9%), Germany (n = 3, 6.7%), France, Malaysia, Italy and Sweden (n = 2, 4.4%) and Spain, Israel, Poland, Finland and Australia (n = 1, 2.2%). The 45 respondents represented 39 organisations; one institution was represented by three respondents, four institutions were represented by two respondents and 34 institutions by a single respondent.

Survey respondents represented the following sectors (multi-sectoral designation was allowed): governmental (n = 26, 58%), public health (n = 25, 56%), research (n = 24, 53%), university (n = 12, 26.7%), food (n = 11, 24.4%), animal (n = 8, 17.8%), private ownership (n = 7, 15.5%), and plant / environment (n = 5, 11.1%). The reported roles of respondents within their institutions (multiple roles were allowed) included: academic / researcher (n = 27, 60%), laboratory scientist / microbiologist (n = 15, 33.3%), bioinformatician (n = 13, 28.9%), public health professional/epidemiologist (n = 10, 22.2%), clinician (n = 3, 6.7%) and infection control practitioner (n = 1, 2.2%). Notably, two respondents identified themselves as post-graduate students (4.4%). Three respondents were representatives of commercial sequencing companies (6.7%) and were excluded from further analysis, which thus included 42 respondents in total.

### Capability and capacity

The majority of respondents had appropriate arrangements in place for shipping microorganisms or DNA (85.7% and 95.2%, respectively) while 64.3% had arrangements for genomic data transfer (Additional file 1: Figure S1). All but one respondent were currently performing NGS and bioinformatics analysis. Internal NGS capability was reported by 84% whereas external access to NGS was reported by 57% (Additional file 1: Figure S2a). Only 14% of respondents were solely dependent upon external NGS services. With regard to bioinformatics, 88% had internal capability whereas only 10% were solely dependent upon external services (Additional file 1: Figure S2b). Forty respondents reported having access to the currently available NGS technologies, consisting of a total of 152 different NGS platforms cumulatively reported. The distribution across NGS technologies is depicted in Additional file 1: Figure S3.

Accessibility to different platforms internally or externally is shown in the Table 1. The three most commonly accessible platforms were MiSeq (23.7%), Ion torrent PGM (15%) and HiSeq 2500 (10.5%). These three platforms accounted for 60.8% of internally accessible sequencers and 30.1% of externally accessible sequencers. Out of 44 NGS platforms available in participating institutions and specifically intended by respondents for use during a PT for NGS, Illumina MiSeq, Ion Torrent PGM and HiSeq 2500 accounted together for 81.8% of instances (24, 9 and 3 out of 44, respectively). The remaining were older HiSeq models (3), PacBio (3), GS 454 FLX (1) and Ion torrent proton (1).

**Table 1:** **Access to NGS platforms as internal or external infrastructure**

**NGS Platform**	**Number having any access**	**Accessible internally**	**Accessible externally**
Ion Torrent PGM	23	15	5
Ion Torrent Proton	6	2	3
GS Junior System (454)	9	5	4
Genome Sequencer FLX (454)	12	8	4
PacBio RS	8	3	5
PacBio RS II	7	3	4
HiScanSQ	3	0	2
HiSeq 1000	4	1	3
HISeq 1500	3	1	2
HiSeq 2000	9	2	7
HiSeq 2500	16	5	8
Genome Analyzer lIx	9	4	4
MiSeq Benchtop Sequencer	36	25	6
ABI SOLiD	6	0	5
other	1	0	1
**Total**	**152**	**74**	**63**

Any information regarding the costs of running NGS using different platforms was provided by 33 out of 42 respondents (78.6%). The reported costs of sequencing a single bacterial genome of 5 MB at coverage 20X and maximum multiplexing were as shown in Additional file 1: Table S1. In the majority of cases (57 out of 75, 76%), sequencing of a single bacterial genome was reported to cost less than US$ 500 at the time of survey. At this cost, sequencing was achieved internally in 48 of 59 platforms (81.3%) as opposing to 9 of 16 platforms externally (56.2%, p = 0.1).

The volume of NGS for bacterial genomes performed annually by respondents is summarized in Additional file 1: Table S2. Of 70 NGS ‘jobs’ reported, 5.7% involved up to 10 genomes and 8.6% over 2,000 genomes. Volume of up to a 100 genomes per year accounted for 75% of external sequencing jobs but only 24.1% of internal sequencing jobs (p < 0.005, OR = 9.46). Sequencing by Illumina technology accounted for 13 / 25 (52%) of jobs involving up to 100 genomes and 31 / 45 (68.9%) of experiments involving over 100 genomes (p = 0.16). Nevertheless, 100% of the 23 NGS ‘jobs’ involving >500 genomes were performed using Illumina technology.

### Sequencing priorities

Information regarding priority pathogens most frequently processed by participating institutions was provided by 34 of 42 respondents. For five categories allowed, a total of 142 pathogens were listed (34 respondents listed at least one category of the most frequently processed pathogen while 23 listed all five priority categories). The taxon distribution of the first category appears in Additional file 1: Figure S4a. Notably, three out four pathogens most frequently sequenced were foodborne pathogens (Additional file 1: Figure S4b). The frequency of taxons sequenced over the passing year by respondents is shown in Additional file 1: Figure S4c. Top 5 sequenced pathogens were again the leading foodborne pathogens and *S. aureus*.

The reasons for using NGS were reported by 41 respondents, according to 11 provided application categories using a 1-5 scale (1 – most important, 5 - least important). The average scores for the 11 categories are shown in Additional file 1: Figure S5a. The leading indication was by far high resolution clustering for outbreak investigation (mean score 1.6) whereas metagenomics, pathogen discovery and evolutionary microbiology were perceived as least important (mean scores >3 points). The consideration in selecting pathogens for using NGS were reported by 41 respondents, according to 9 provided application categories using a 1-5 scale (1 – most important, 5 - least important). The average scores for the 11 categories are shown in Additional file 1: Figure S5b. The leading consideration was by far a high impact on public health (mean score 1.69) followed by utility for performing real time laboratory surveillance (mean score 2.32).

### Attitudes towards proficiency testing for NGS

None of the 41 respondents strongly disagreed with any of the nine statements concerning the evaluation criteria for PT for NGS (Table 2). Over 75% of respondents expressed agreement or strong agreement with all nine statements. In particular, accurate classification of existing frequently tested and globally relevant pathogens (e.g., foodborne *Salmonella*) as well as phylogenetic tree building were statements with which >90% agreed or strongly agreed. Any disagreement was noted in six out of the nine statements but at a rate below 10%.

**Table 2:** **Agreement with possible evaluation criteria of PT for NGS**

**Statement**	**Strong disagreement** **Strong agreement**					**% agreement**
17.1 Assessment of the quality of WGS reads is a very important consideration	0	2	4	15	20	85.4%
17.2 Ability to integrate and accommodate sequence data from multiple vendor platforms is a very important consideration	0	2	4	17	18	85.4%
17.3 Capacity for de novo sequencing and genome assembly is a very important consideration	0	3	6	21	11	78%
17.4 Capacity for analysis of emerging biothreats is a very important consideration	0	0	11	19	11	73.2%
17.5 Accurate classification of existing frequently tested and globally relevant pathogens (e.g., foodborne *Salmonella*) is a very important consideration	0	0	3	22	16	92.7%
17.6 Quality of reference based assembly is a very important consideration	0	3	6	20	12	78%
17.7 Quality of annotation is a very important consideration	0	2	8	24	7	75.6%
17.8 Single nucleotide polymorphism (SNP) calls is a very important consideration	0	2	4	15	20	85.4%
17.9 Tree building is a very important consideration	0	0	4	24	13	90.2%

### Operational aspects of PT for NGS

**Figure 1:**
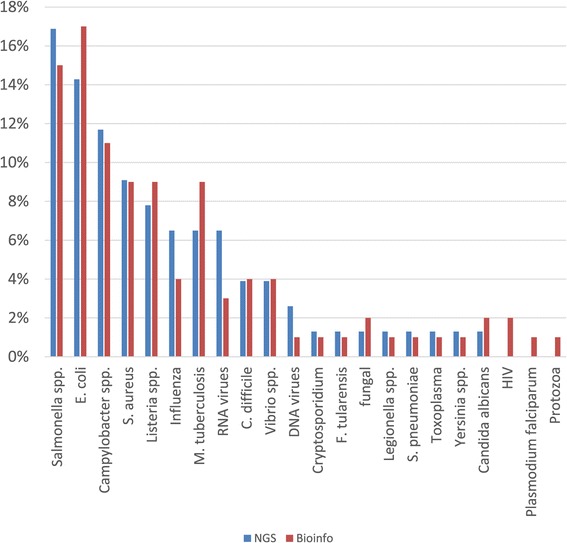
**Priority pathogens for inclusion in PT for NGS sequencing and NGS bioinformatics analysis.**

Respondents were asked to delineate five priority pathogens for inclusion in the PT for NGS that will look at all stages of sequencing and analysis processes. Based on 24 respondents providing this information, *Salmonella* was by far the top priority for NGS PT, listed by 9 respondents, followed by *S. aureus* and RNA viruses (3 each), *L. monocytogenes*, *M. tuberculosis* and *E. coli* (2 each) and influenza virus, *Campylobacter spp*. and *C. difficile*, (1 each). When prioritisation was generated after pooling all five priority categories reported (Figure 1), the leading pathogens were *Salmonella* (17%), *E. coli* (14%) and *Campylobacter spp*. (12%), followed by *S. aureus* (9%) and *L. monocytogenes* (8%). Respondents were also asked to delineate five priority pathogens for inclusion in a PT for NGS carried out by provision of simulated datasets for bioinformatics analysis. Based on 26 respondents providing this information, *Salmonella* was again the top priority for NGS PT, listed by 9 respondents, followed by *S. aureus* (4), *E. coli* (3), RNA viruses, *L. monocytogenes*, *M. tuberculosis* and *Enterobacteriaceae* (2 each) and influenza virus and *Campylobacter spp*. (1 each). When prioritisation was generated after pooling all five priority categories (Figure 1), the leading pathogens were *E. coli*, *Salmonella* and *Campylobacter spp*. followed by *L. monocytogenes*, *M. tuberculosis* and *S. aureus*.

With regards to the number of different strains to be used in the PT, per dispatch, 36 respondents displayed the following preferences (Additional file 1: Table S4): 39.4%, 57.6% and 53.1% of respondents regarded PT samples containing viruses, fungi and protozoa as not relevant, as compared to only 8.3% for bacterial PT. A substantial proportion of respondents were willing to process 4 bacterial PT samples per dispatch (44.4% for strains, 47.2% for DNA and 50% for genomic datasets). Of those interested in viral PT, 60% were willing to process 4 samples per dispatch.

### NGS and bioinformatics practices

**Figure 2:**
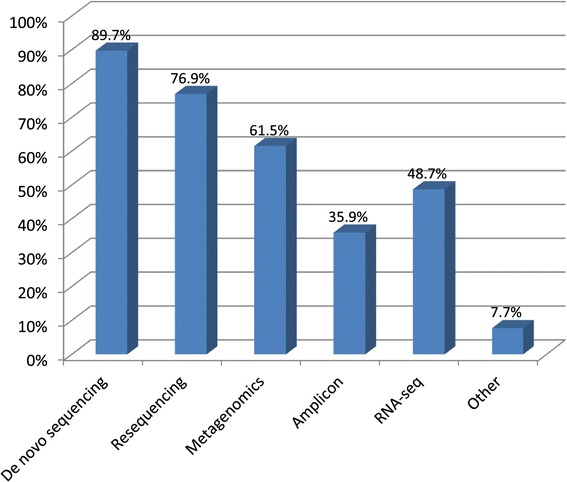
**Intended use of NGS data.**

The survey of current technical NGS and bioinformatics practices and usage included a series of 15 questionnaire items with a varying response rate. The intended use of NGS data were reported by 39 respondents and is shown in Figure 2. NGS was used commonly for *de novo* sequencing, resequencing, metagenomics and RNA sequencing. Two respondents highlighted data would be used to create public health policy or develop bioinformatics tools. For library preparation, a notable diversity in methods used was reported among 39 respondents with transposon-based fragmentation being the most common method, followed by physical shearing and enzymatic shearing (Additional file 1: Figure S6). In addition, 74.3% reported multiplexing of samples in NGS runs was being performed. Notably, 29% reported not to be performing hands-on library preparation. Importantly, only 46.7% of respondents (35.9% overall) were routinely including standard or reference materials in their NGS runs.

The commonly expected coverage while performing NGS for bacteria was 31-60X (51.3% of respondents) and coverage of 11-30X or over 60X was reported by 21.6% and 18.9%, respectively (Additional file 1: Table S5). Of those performing NGS for viruses, 12 out of 17 (76%) were working at coverage of >60X. Very few were performing practical NGS for fungi and protozoa, with results varying.

**Figure 3:**
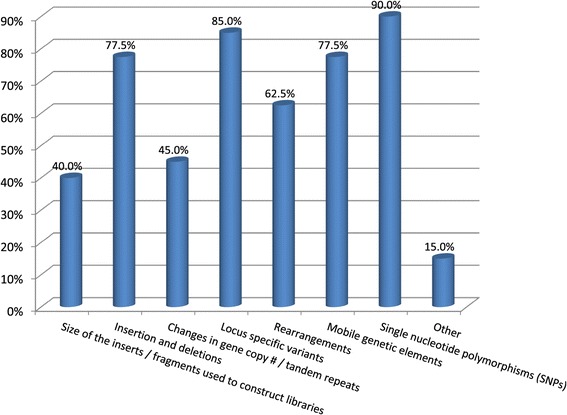
**Genomic information intended to be captured from NGS.**

The genomic information intended to be captured from NGS data analysis was diverse (Figure 3). Single nucleotide polymorphisms (SNPs) and locus-specific variations were the most commonly expected outputs of NGS analysis (90% and 85%, respectively), followed by mobile genetic elements and insertions/deletions (indels) (77.5% each). Of 29 respondents, 86.2% reported low-quality base trimming during bioinformatics analysis. Those few not performing trimming reported it was either not necessary for their intended use, performed automatically by their NGS software or will consider trimming in future analyses.

The vast majority of respondents perceived quality filtering as important to any extent (92%) and 56% very important (Additional file 1: Figure S7). In addition, of 34 respondents performing assemblies of NGS data, 73.5% reported having any established criteria for quality assessment and quality control of assemblies. Most of the 25 respondents reporting having quality criteria for assemblies in place employed more than one criterion. The frequency of various criteria is shown on Additional file 1: Figure S8. The most commonly used criterion was coverage (90.9%) followed by number of bases and mapping of reads to reference (68.2% each). When respondents were asked to provide values for quality criteria, a wide variety of responses was noted and no conclusions could be drawn.

The use of bioinformatics software is shown in Additional file 1: Figure S9. Of 35 respondents, 71% stated they used mainly or exclusively externally developed software whereas 23% relied mainly but not exclusively on internally developed software. In 6% analyses were done elsewhere. Of those 35, 32 respondents also provided information regarding specific assembly software being used. The leading software was Velvet (75%), Newbler (46.9%) and CLC (46.9%) and SOAPdenovo (25%). Other software used by less than 20% of users included ABySS, ALLPATHS-LG, CABOG, Edena, Euler, Mira, MSR-CA, SGA, SSAKE, VCAKE, SPAdes, Cortex, CloVR, RAST, Geneious and SAMtools.

Thirty respondents provided information regarding specific mapping software packages being used. The leading software was BWA (66.7%), Bowtie 2 (53.3%) and Bowtie 1 (23.3%). Other software used by 10% of users included Novoalign and SMALT, while BFAST, MAQ, SHRiMP, SSAHA2, tmap and Geneious were used by less than 10%.

## Discussion

The WGS data is worth more if it is shared globally in an open source manner and linked to clinical and epidemiological contexts (e.g., informative metadata). Notably, pioneering studies to inform implementation of NGS based real-time prospective surveillance and analysis of foodborne pathogens such as *Listeria monocytogenes* and *Salmonella enterica* are underway in leading institutions throughout North America and Europe. Moreover, the use of NGS for near real-time investigation of probable transmission pathways has already been reported [6,17-19].

As WGS is applied to public health surveillance, standardising quality metrics becomes critical. These metrics include, for example, standards for calibration, validation, and comparison among platforms; data reliability, robustness, and reproducibility, and the quality of assemblers [3,16]. Like any technology, WGS has its advantages and limitations. Potential uncertainties and errors can be introduced into the sequence analysis by the sequencing machines, analytical algorithms and residual errors in the reference data with what we align the new sequence. Thus proficiency testing programs that cover both sequencing “wet lab” and analytical “dry lab” steps are urgently required. To our knowledge, this is the first review of the current state of play, needs and priorities in relation to the proficiency testing for WGS performed in the field of microbiology.

### Key findings

The current report illustrates current NGS and bioinformatics capability and practice within the GMI community and attitudes towards the setting up and delivery of a PT programme for NGS. Our survey highlights the professional diversity of individuals engaged in NGS-based projects and the wide range of capabilities within institutions. For example, some institutions are currently performing NGS on a limited basis, mainly by relying on external sequencing and analysis services, while other institutions are running large scale NGS studies with internal sequencing and computational infrastructures. This diversity is also associated with a notable range of costs per sample.

The priority pathogens reported by respondents that are being investigated with NGS represent the entire gamut of foodborne illness, with emphasis on the pathogens associated with highest disease burden in humans, followed by ‘high profile’ non-foodborne pathogens of clinical and public health importance such as *M. tuberculosis*, *S. aureus* and RNA viruses. This is in agreement with the fact that key outputs expected from NGS are of use in molecular epidemiology, high resolution typing and outbreak investigation.

For most end-users, the performance of and participation in PT was perceived as important. Information collated through this survey will help guide the PT in terms of the pathogens included, PT frequency and technical requirements. The wide range of sequencing and bioinformatics practices reported by end-users highlights the importance of standardization and harmonisation of NGS in public health and underpins the use of PT as a means to assuring quality.

### Quality consideration for NGS in microbiology

There are significant differences in the sequencing methods, specimen preparation, run throughput and hands-on time between different sequencing platforms. In addition, the amount of sequencing data sufficient for pathogen characterisation (i.e. genome ‘coverage’) and associated outbreak investigations remains the subject of debate [1,20]. These variables may have technology- and coverage-specific effects on the detection of genomic variants. Thus laboratories are expected to balance the pathogen genome characteristics, the instrument throughput, the accuracy of variant-calling algorithms and the cost of sequencing runs.

The outputs generated by different sequencing platforms are subjected to multiple analytical steps that usually start with sequence assembly or reference-based mapping and finish with simultaneous comparisons of multiple genomes and data visualisation. Bioinformatic approaches for genome-wide analyses of pathogens are highly varied across the microbiology community, with an abundance of tools continually being developed, refined and packaged together as software ‘pipelines’ [21].

Whilst NGS based surveillance is expected to become common in the near term [3], identification of pathogens, rather than traceback investigations, is likely to be among the last areas where NGS becomes routine as the cost are high and other technologies such as qPCR and MALDI-TOF are effective. However, the technology could be employed to detect yet unknown, emerging or fastidious pathogens. Furthermore, deep sequencing would allow in the near future, identification of pathogens from primary clinical samples and/or to characterize the normal microbiota and pathogenic flora of non-sterile body sites using meta-genomic strategies.

### Impact of survey outputs on envisaged PT for NGS

Principles of NGS standardisation for clinical testing have been recently outlined by a national working group on laboratory medicine convened by the US Centers for Disease Control and Prevention [16]. Their recommendations emphasise the need for adequate validation, quality control, use of reference materials and performance of independent PT. In agreement with such recommendations, GMI is currently executing a pilot PT scheme with intended full roll-out in ultimo 2014. The main objective of this PT is to ensure harmonisation and standardisation in whole genome sequencing and data analysis, with the aim to produce comparable data for the GMI initiative. A further objective is to assess and improve the uploaded data to databases such as NCBI, EBI and DDBJ. Therefore, the laboratory work analysis performed for this PT should be done by using the methods routinely used in the individual laboratories.

The PT will consist of two wet-lab and one dry-lab component(s) targeting priority microorganisms such as *Salmonella*, *E. coli* and *S. aureus*. The PT will emphasise NGS applications in microbiology highlighted by the survey (e.g. SNP analysis). The wet-lab components to be provided, will assess the laboratories ability to perform DNA preparation, sequencing procedures and analysis of epidemiological markers whereas the dry component will assess the laboratories’ ability to analyse a whole-genome-sequencing dataset and distinguish between clonally related and sporadic genomes. In order to achieve this, the PT substrates will be provided to participants via transport of lyophilised live cultures and stabilized bacterial DNA distributed by courier and electronic *fastq* datasets provided through ftp servers. All PT stages will follow standardised procedures.

### Study limitation

These conclusions should be interpreted in light of the limitations in the study design. First, the survey relied on self-reported behaviour without verification that participants actually practiced in the manner described. Second, we surveyed only GMI participants. Scientists in the developing world are likely to differ from developed world practitioners in their technology use and in information needs. However, this has the advantage of representing the point of view of professionals who are usually “early adopters” of new concepts and the opinion leaders in their field. Third, there may be a volunteer bias related to the fact the those community member being more advanced in NGS implementation or having increased interest in moving into NGS were more likely to sign up to the survey.

## Conclusions

The significant variation in the use of NGS and data analytics in public health microbiology and differences in attitudes of microbiologists deserve careful consideration. Important for the reliability of submitted sequence data to a GMI database and other public sequence archives will be the test of the congruence of outputs among members’ in DNA extraction, library preparation, the actual sequencing, assembly and phylogenetic analysis following different laboratory protocols, software tools, and platforms [16,21]. GMI aims to assist laboratories and partners globally to perform NGS to the highest degree of quality. The findings of our survey will guide the PT activities of the GMI to ensure it meets the expectations of the end-users. In addition we have gathered information on capability, attitudes and practices of GMI community members. It is envisaged that PT of WGS in microbiology will be a dynamic process that will continuously evolve and, thereby, inform the introduction of NGS into clinical and public health microbiology practice and will inevitably become the routine tool for external quality assurance in the post-genomic era.

## Additional file

Additional file 1:
**Table S1.** **Costs of bacterial NGS by platform as reported by respondents at time of survey.** **Table S2.** Volume of bacterial NGS performed annually by respondents. **Table S3.** Preferred number of strains per dispatch of PT for NGS. **Table S4.** Expected coverage while performing NGS for various taxa*. **Figure S1.** Organism and data transfer arrangements in place. **Figure S2a.** NGS capability of participating institutions. **Figure S2b.** Bioinformatics capability of participating institutions. **Figure S3.** Distribution of NGS access across technologies. **Figure S4a.** Distribution of top priority pathogens most commonly processed in participating institutions. **Figure S4b.** Distribution of pathogens most commonly processed in participating institutions. **Figure S4c.** Frequency of taxons genome-sequenced over passing year. **Figure S5a.** Main purpose of NGS experiments (mean scores). **Figure S5b.** Criteria for selecting pathogens for NGS experiments (mean scores). **Figure S6.** Library preparation methods employed for NGS. **Figure S7.** Perception of the importance of quality filtering during NGS analysis. **Figure S8.** Frequency of criteria used for QA/QC of assemblies. **Figure S9.** Use of bioinformatics software for analysis.

## Abbreviations

NGS: Next-generation sequencing
WGS: Whole genome sequence
MLST: Multi-locus sequence typing
PFGE: Pulsed-field gel electrophoresis
GMI: Global Microbial Identifier
WG: Working Group
PT: Proficiency testing
