# Supplementary material for: Proficiency testing for bacterial whole genome sequencing: an end-user survey of current capabilities, requirements and priorities
Source: BMC Infect Dis. 2015 Apr 3;15:174. doi: 10.1186/s12879-015-0902-3 (PMC4392855; doi:10.1186/s12879-015-0902-3)

Table S1. Costs of bacterial NGS by platform as reported by respondents at time of survey

Sequencing platform	<US$ 100	US$ 101-500	US$ 501-1000	>US$ 1000
Ion Torrent PGM internal	0	11	0	0
Ion Torrent PGM external	1	1	0	0
Ion Torrent Proton	No data provided			
GS Junior System (454)	No data provided			
GS FLX (454) internal	0	0	2	2
GS FLX (454) external	0	0	0	1
PacBio RS internal	0	0	3	1
PacBio RS external	0	0	0	1
PacBio RS II internal	0	2	2	0
HiScanSQ external	1	0	0	1
HiSeq 1000 external	0	0	0	1
HiSeq 1500	No data provided			

HiSeq 2000 internal	1	2	0	0
HiSeq 2000 external	0	1	1	0
HiSeq 2500 internal	4	3	0	0
HiSeq 2500 external	1	0	0	1
GA IIx internal	1	2	0	0
MiSeq Benchtop internal	9	13	1	0
MiSeq Benchtop external	1	3	0	1
ABI SOLiD	No data provided			
Other	No data provided			
**Total internal**	**15**	**33**	**8**	**3**
**Total external**	**4**	**5**	**1**	**6**
**Grand total**	**19 (25.3%)**	**38 (50.7%)**	**9 (12%)**	**9 (12%)**

Table S2. Volume of bacterial NGS performed annually by respondents (Q10)

Sequencing platform	1-10	11-100	101-500	501-2,000	>2,000
Ion Torrent PGM internal	0	3	6	0	0
Ion Torrent PGM external	2	1	0	0	0
Ion Torrent Proton	No data provided				
GS Junior (454)	No data provided				
GS FLX (454) internal	0	0	1	0	0
GS FLX (454) external	0	0	0	0	0
PacBio RS internal	0	2	2	0	0

PacBio RS external	0	1	0	0	0
PacBio RS II internal	0	3	1	0	0
HiScanSQ external	0	0	1	0	0
HiSeq 1000 external	0	1	0	0	0
HiSeq 1500	No data provided				
HiSeq 2000 internal	0	1	0	2	0
HiSeq 2000 external	0	2	0	0	1
HiSeq 2500 internal	0	0	1	3	3
HiSeq 2500 external	0	1	0	0	0

GA IIx internal	1	0	0	2	0
MiSeq Benchtop internal	1	2	9	10	1
MiSeq Benchtop external	0	4	1	0	1
ABI SOLiD	No data provided				
Other	No data provided				
**Total internal**	**2**	**11**	**20**	**17**	**4**
**Total external**	**2**	**10**	**2**	**0**	**2**
**Grand total**	**4 (5.7%)**	**21 (30%)**	**22 (31.4%)**	**17 (24.3%)**	**6 (8.6%)**

Table S3. Preferred number of strains per dispatch of PT for NGS

PT sample type	1	2	3	4	N/A
24.1 Microorganisms (bacterial) for DNA purification and sequencing	4	4	9	16	3
24.2 Microorganisms (viral) for DNA purification and sequencing	2	2	4	12	13
24.3 Microorganisms (fungal) for DNA purification and sequencing	4	1	5	4	19
24.4 Microorganisms (protozoan) for DNA purification and sequencing	4	4	3	4	17
24.5 Samples of DNA for sequencing	2	6	6	17	5
24.6 Datasets from NGS platforms for data analysis	2	3	9	18	4

N/A – not applicable

Table S4. Expected coverage while performing NGS for various taxa*

	less than 10	11-30	31-60	over 60	Not applicable
Bacteria	1	8	19	7	2
Virus	0	3	2	12	14
Fungus	0	5	1	1	23
Protozoa	1	4	0	3	21

*coverage may be considered platform-dependent

Figure S1. Organism and data transfer arrangements in place

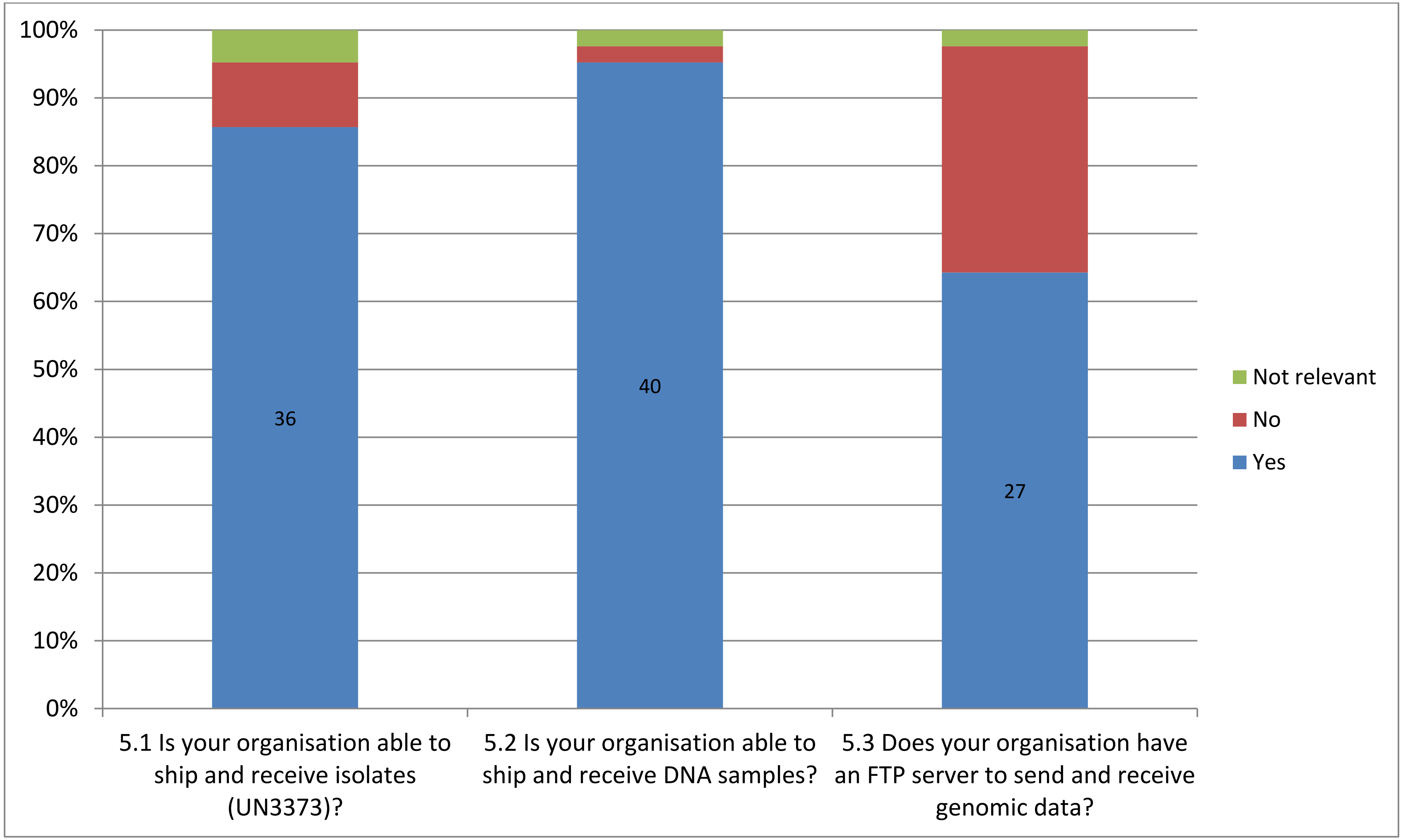

Figure S2a. NGS capability of participating institutions

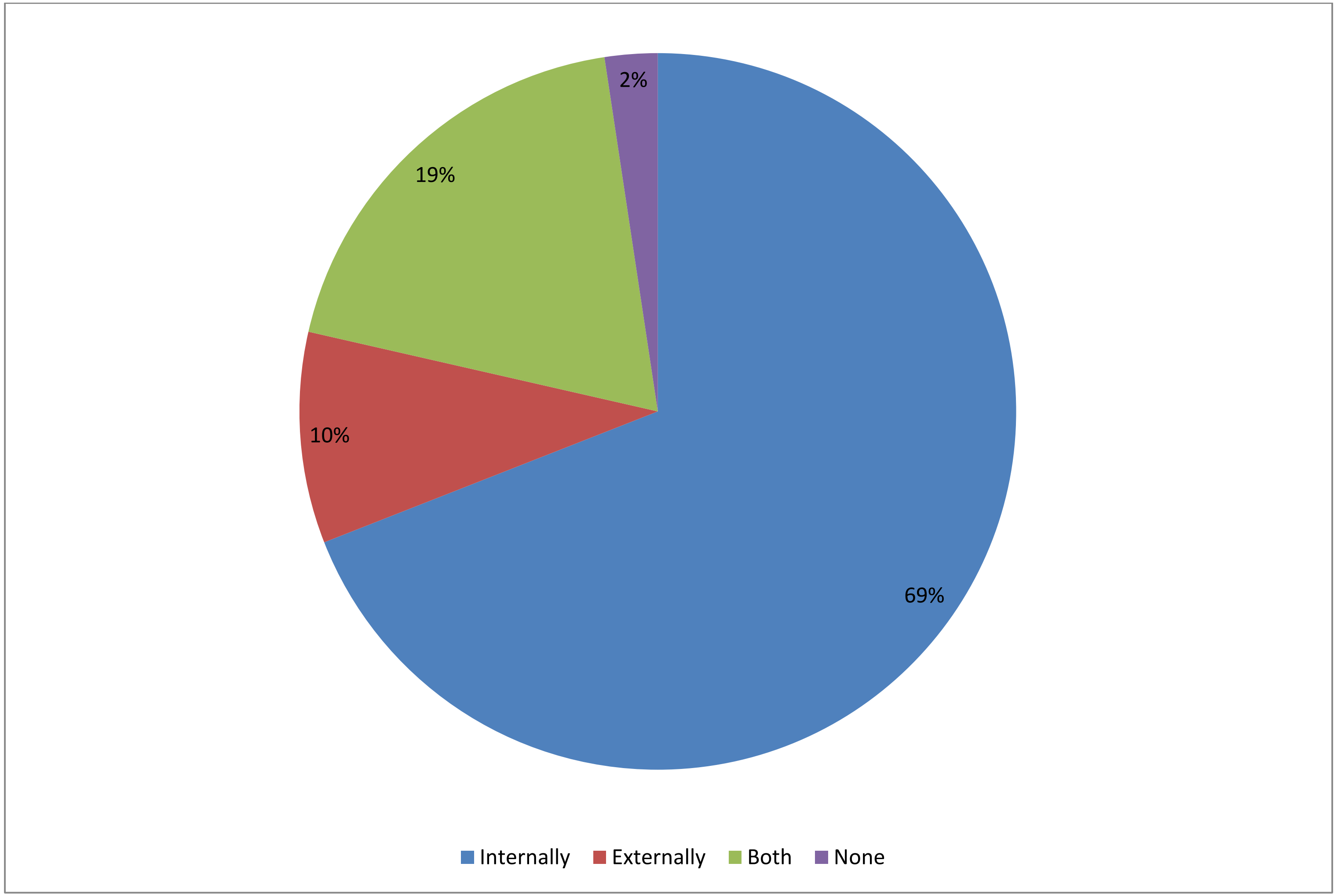

Figure S2b. Bioinformatics capability of participating institutions

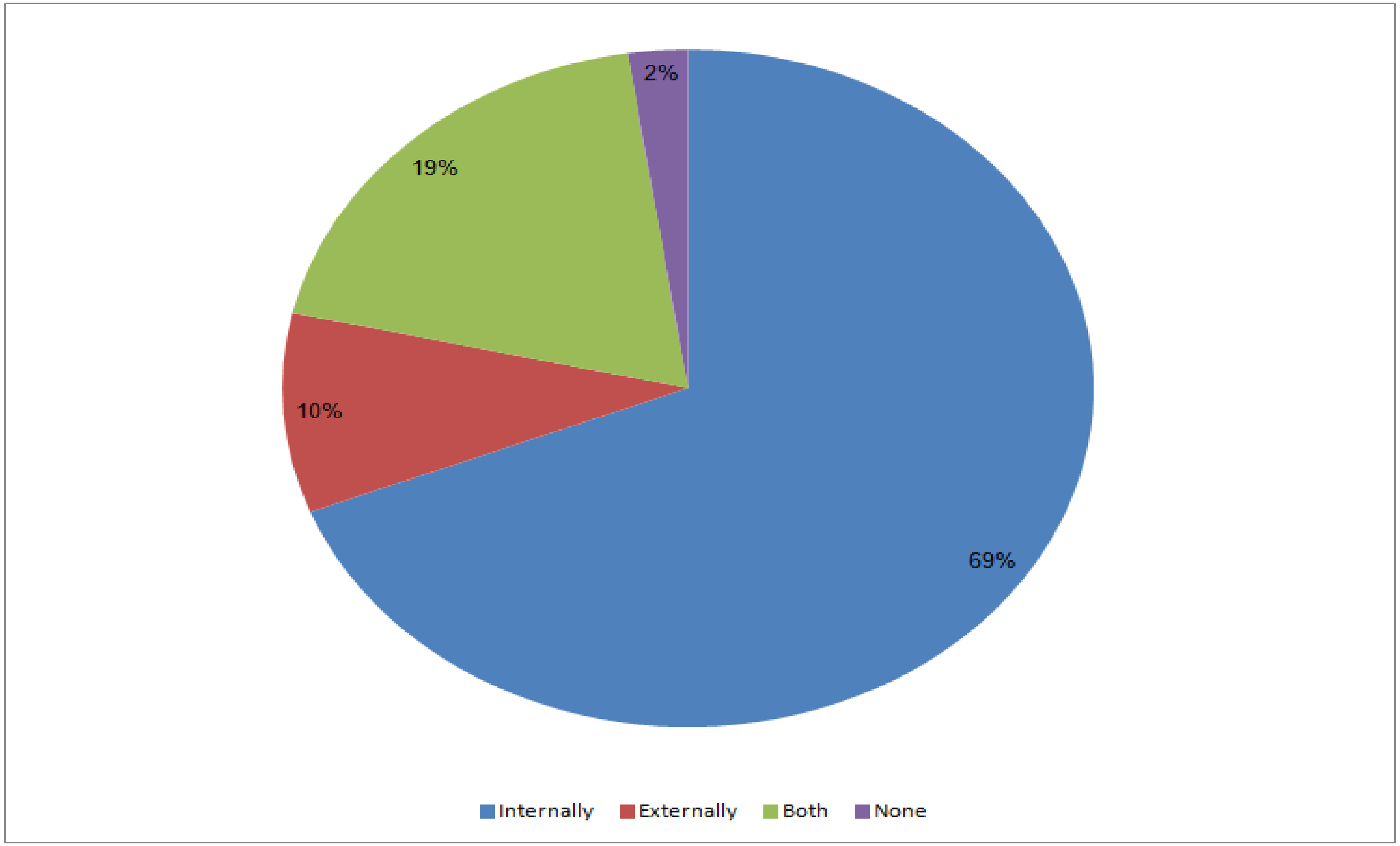

Figure S3. Distribution of NGS access across technologies

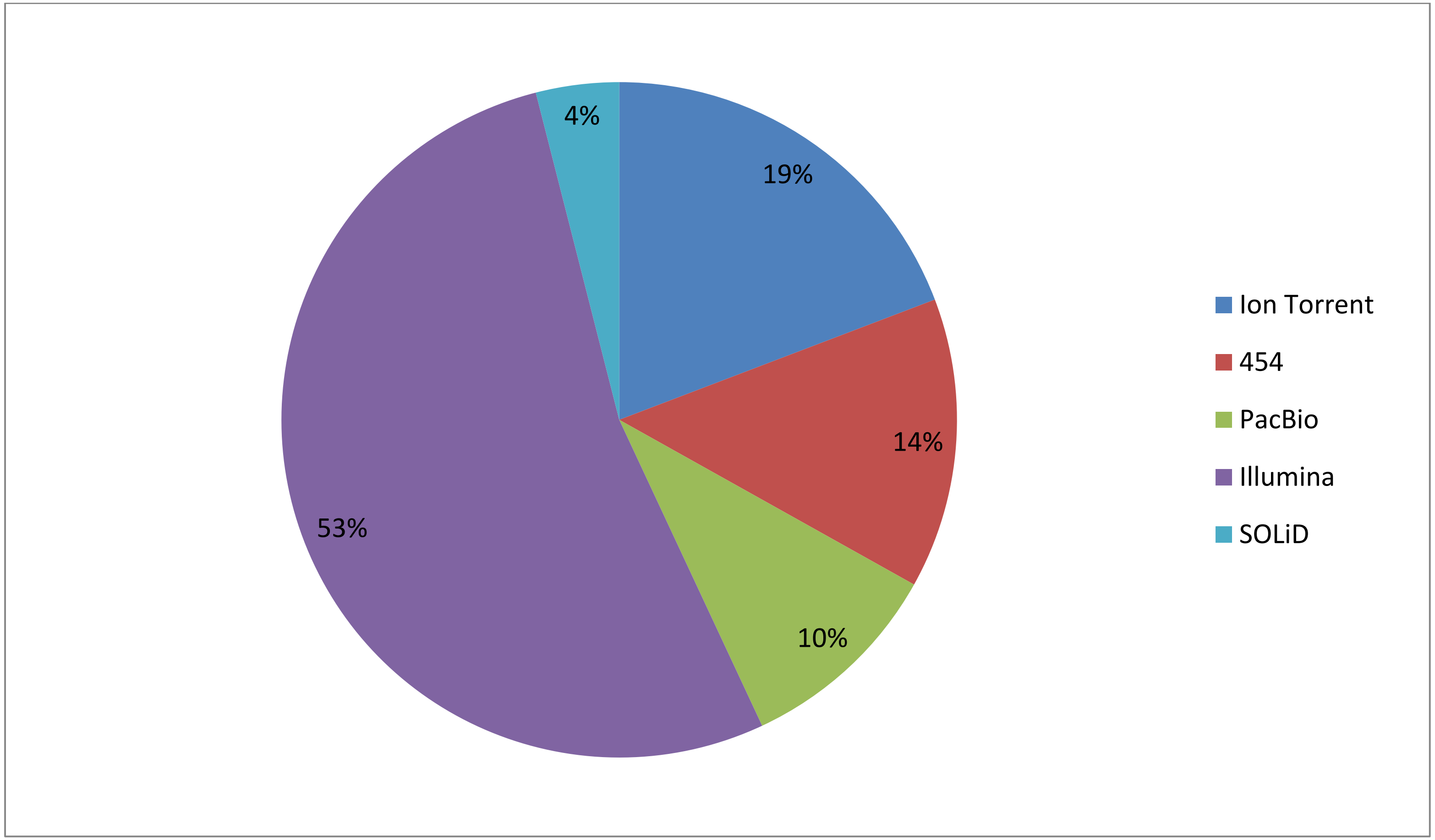

Figure S4a. Distribution of top priority pathogens most commonly processed in participating institutions

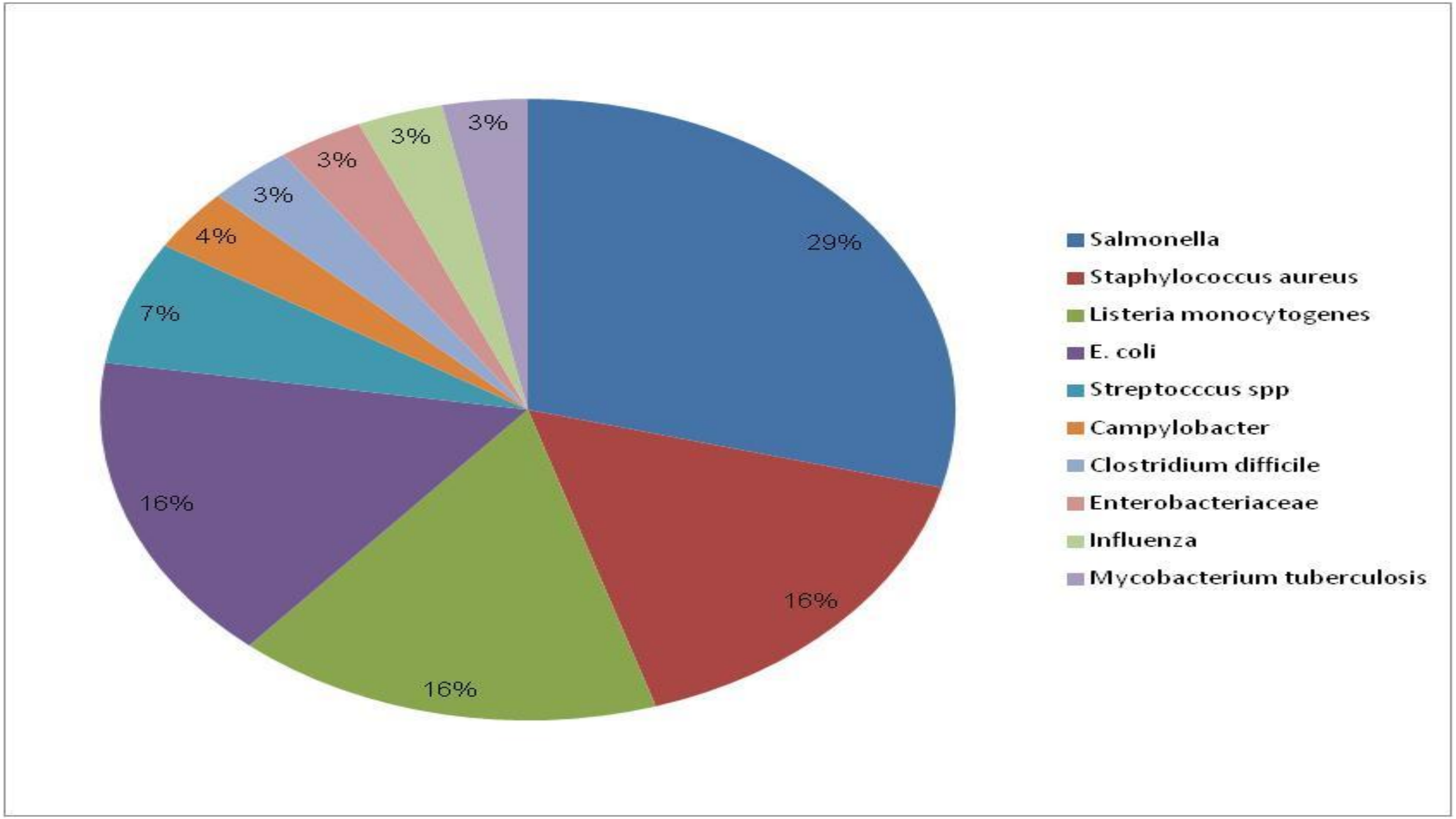

Figure S4b. Distribution of pathogens most commonly processed in participating institutions

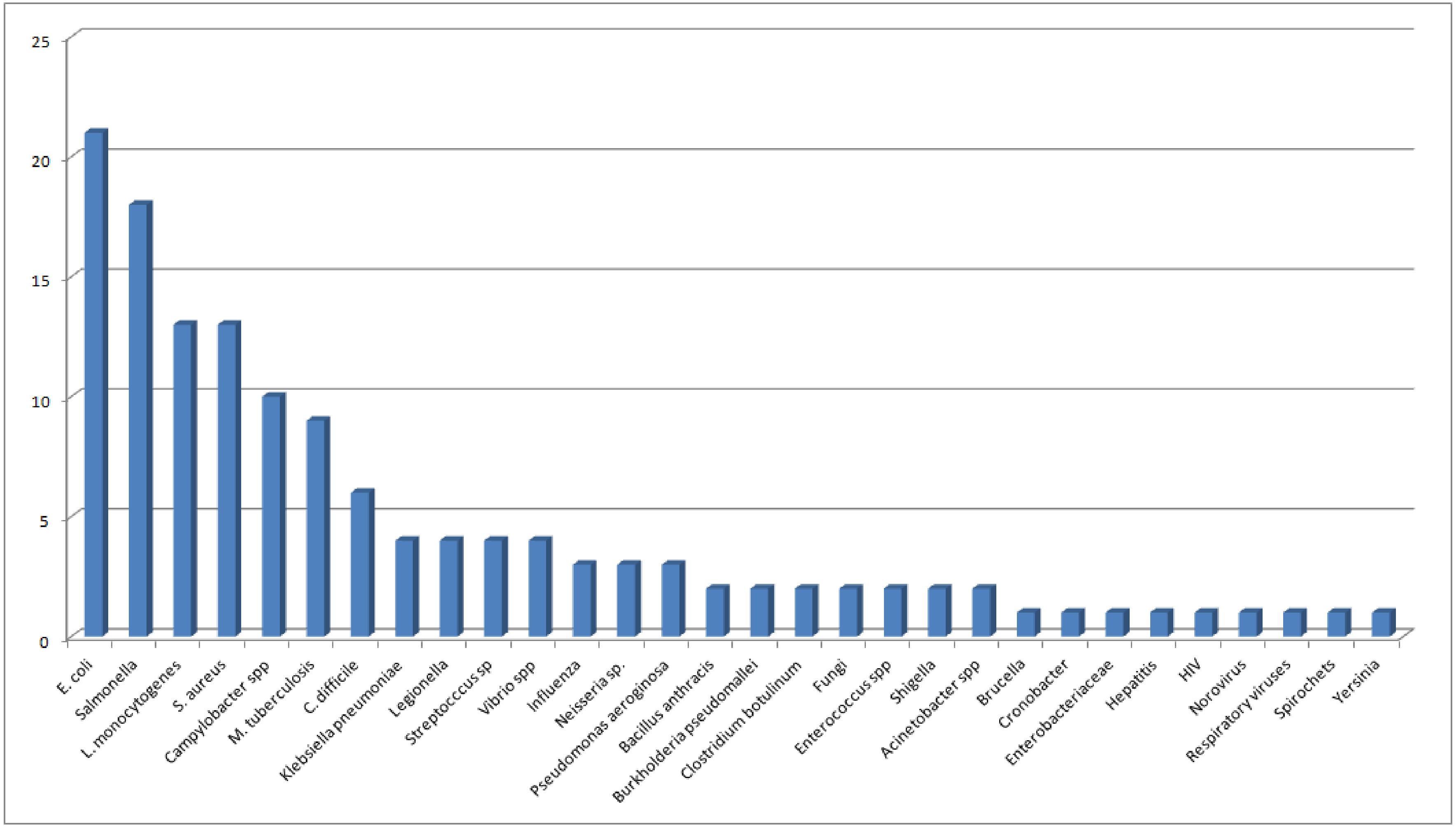

Figure S4c. Frequency of taxons genome-sequenced over passing year

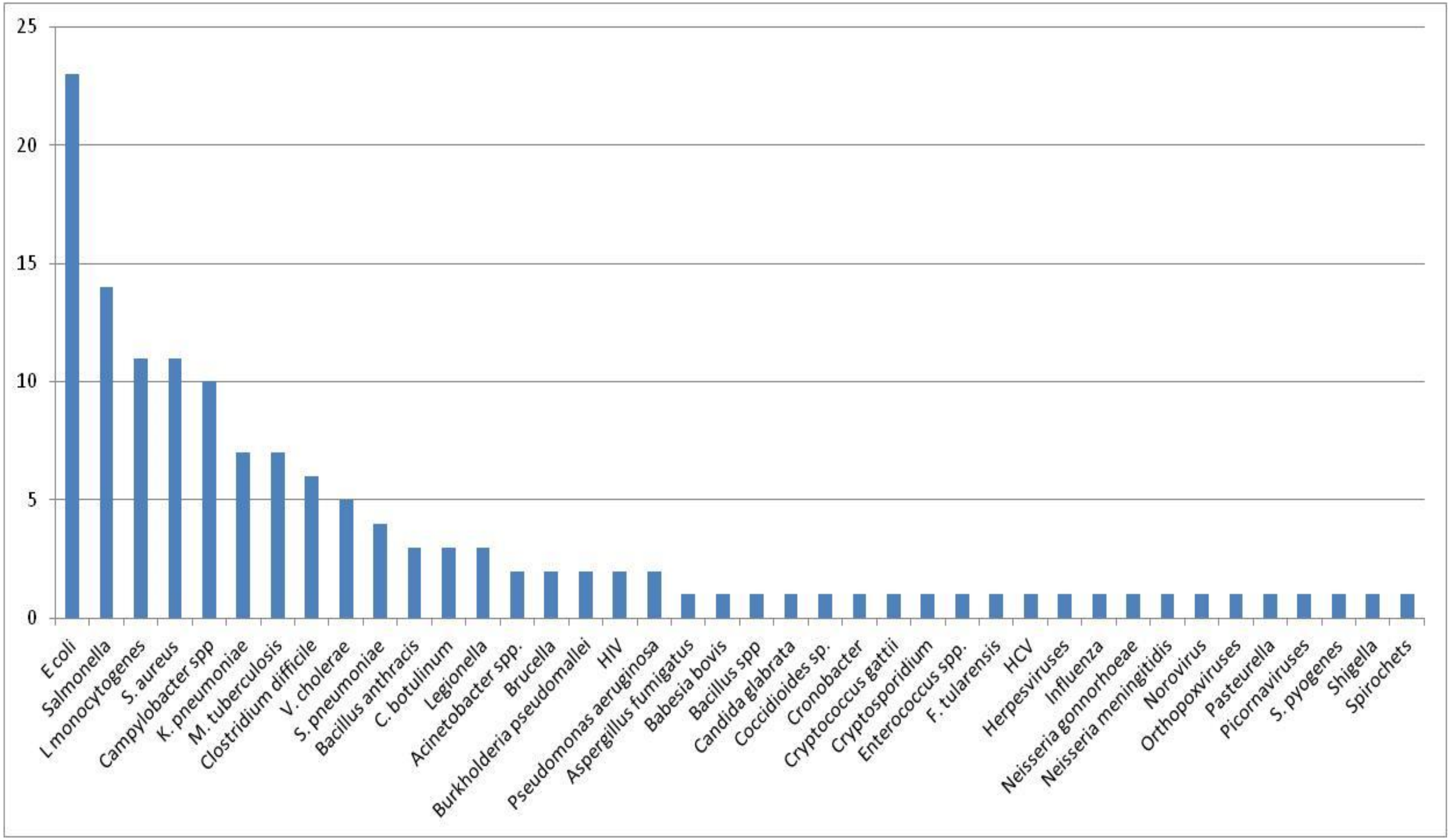

Figure S5a. Main purpose of NGS experiments (mean scores)

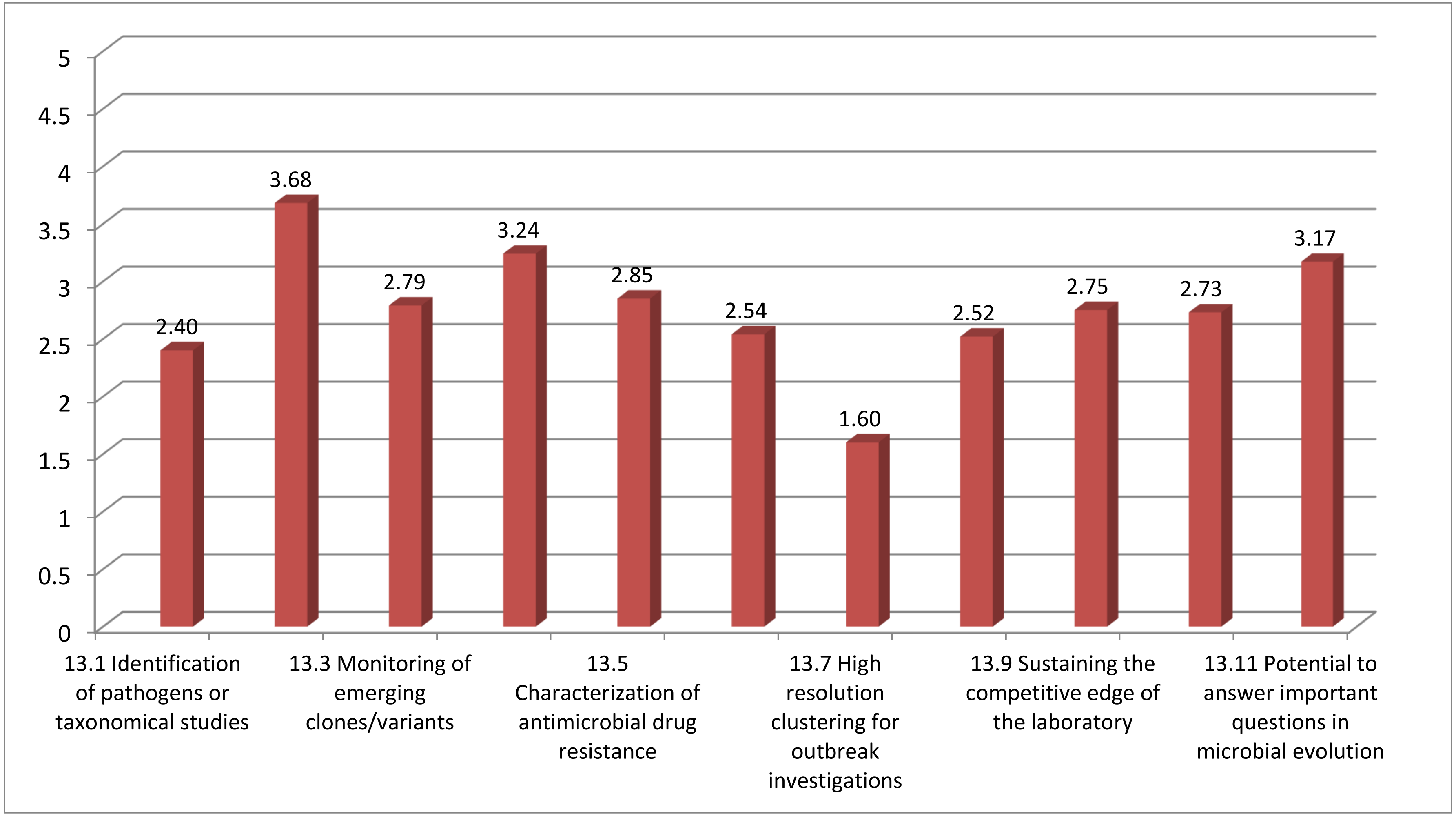

Figure S5b. Criteria for selecting pathogens for NGS experiments (mean scores)

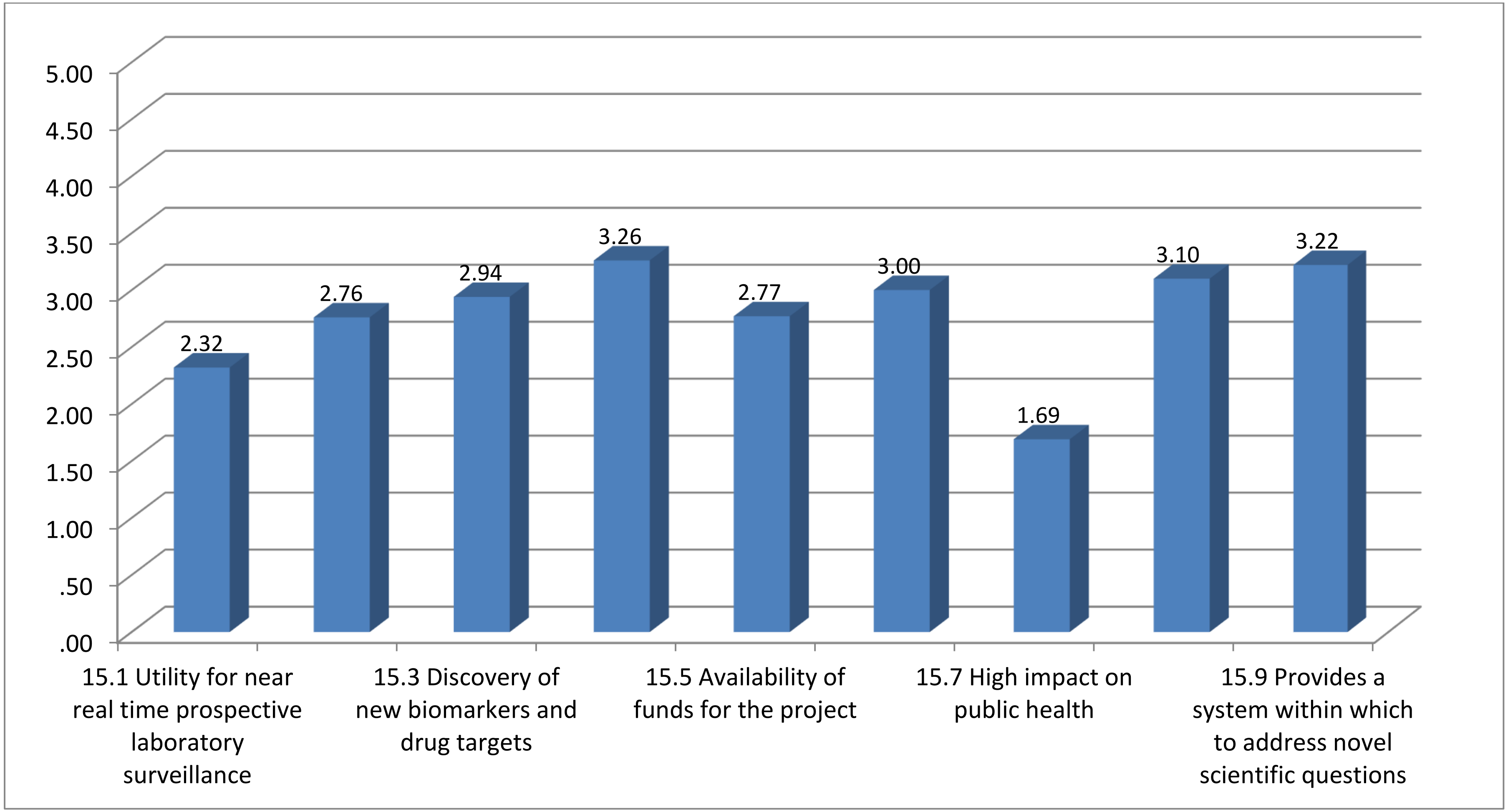

Figure S6. Library preparation methods employed for NGS

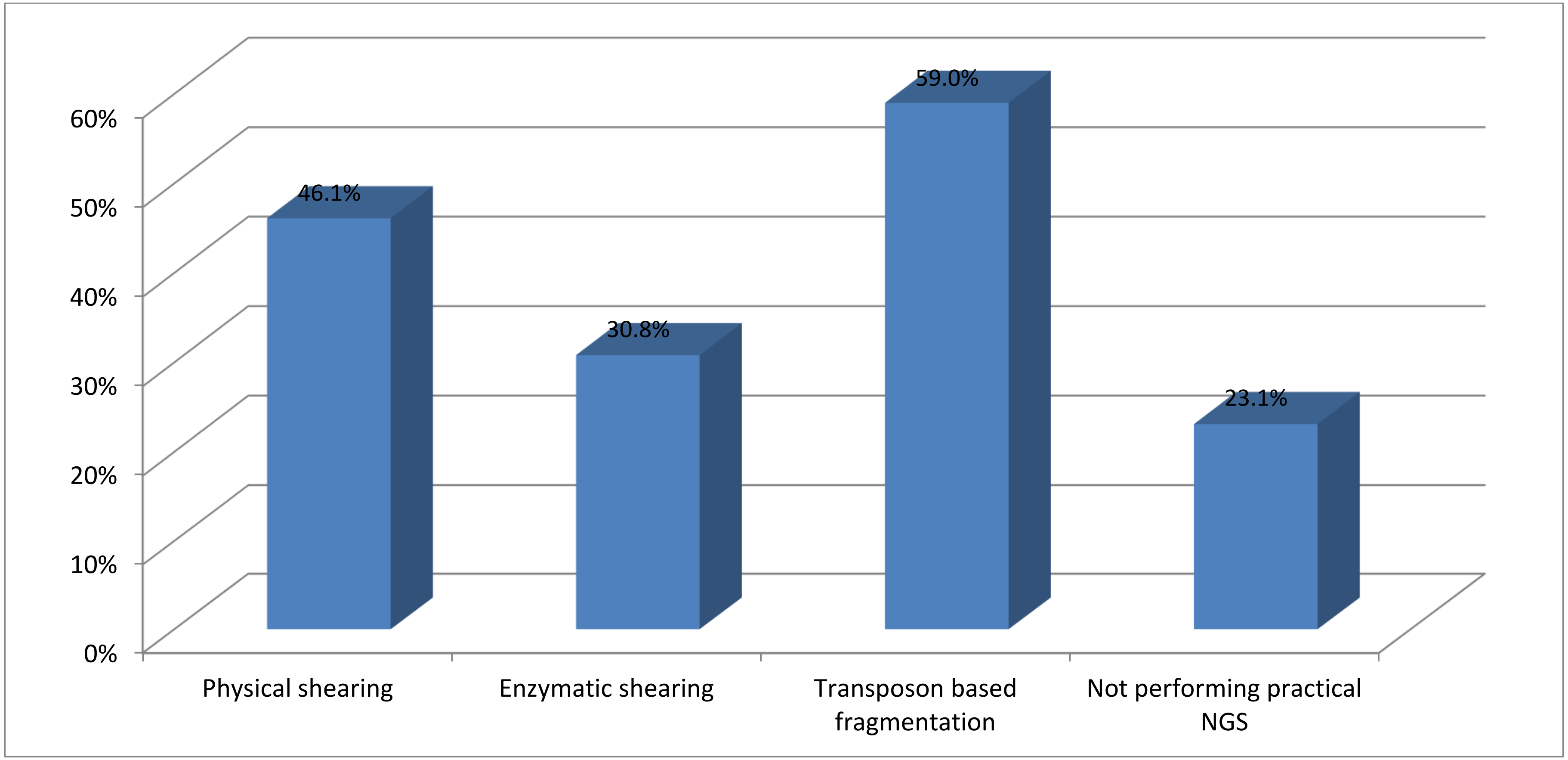

*coverage may be considered platform-dependent

Figure S7. Perception of the importance of quality filtering during NGS analysis

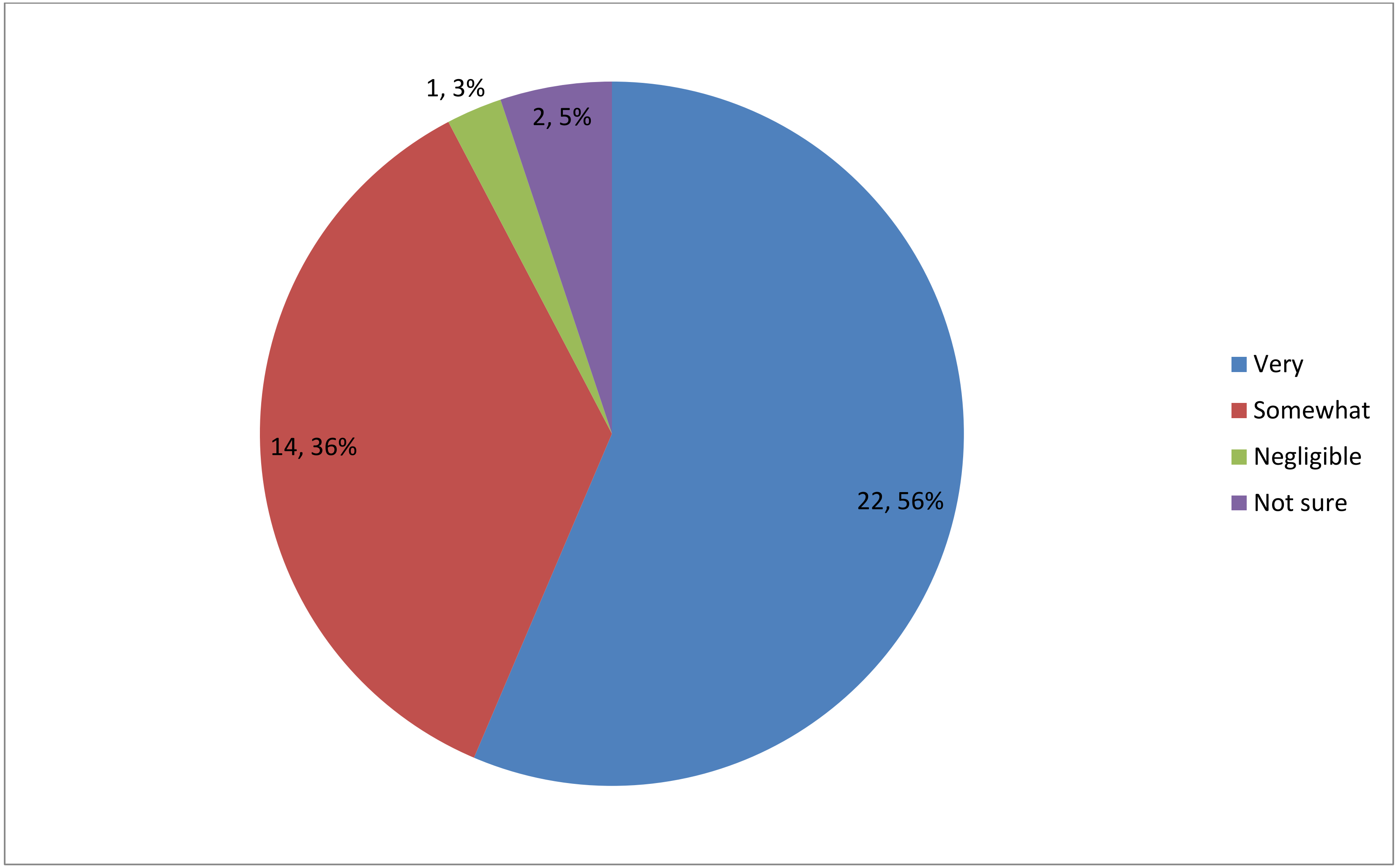

Figure S8. Frequency of criteria used for QA/QC of assemblies

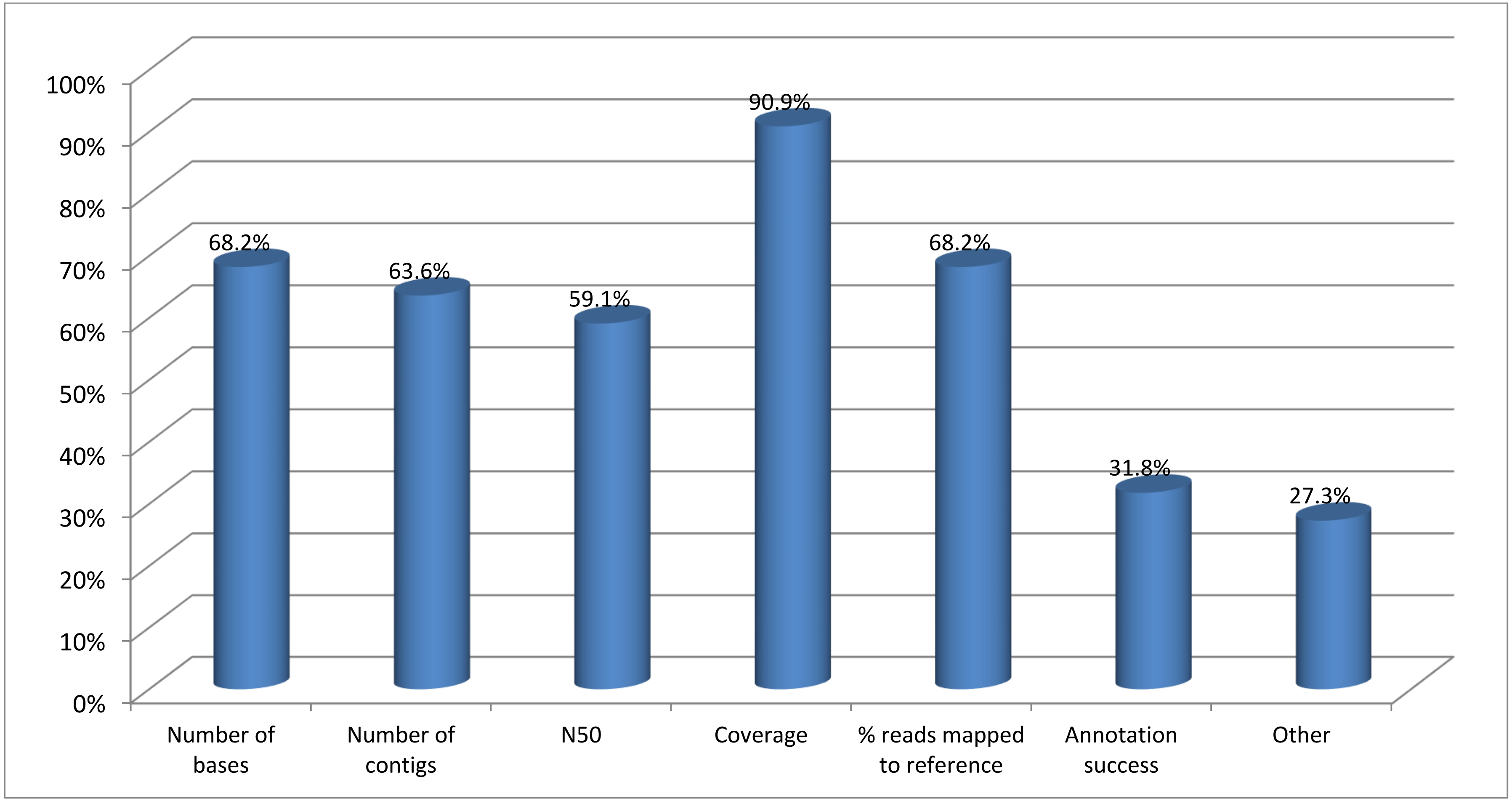

Figure S9. Use of bioinformatics software for analysis

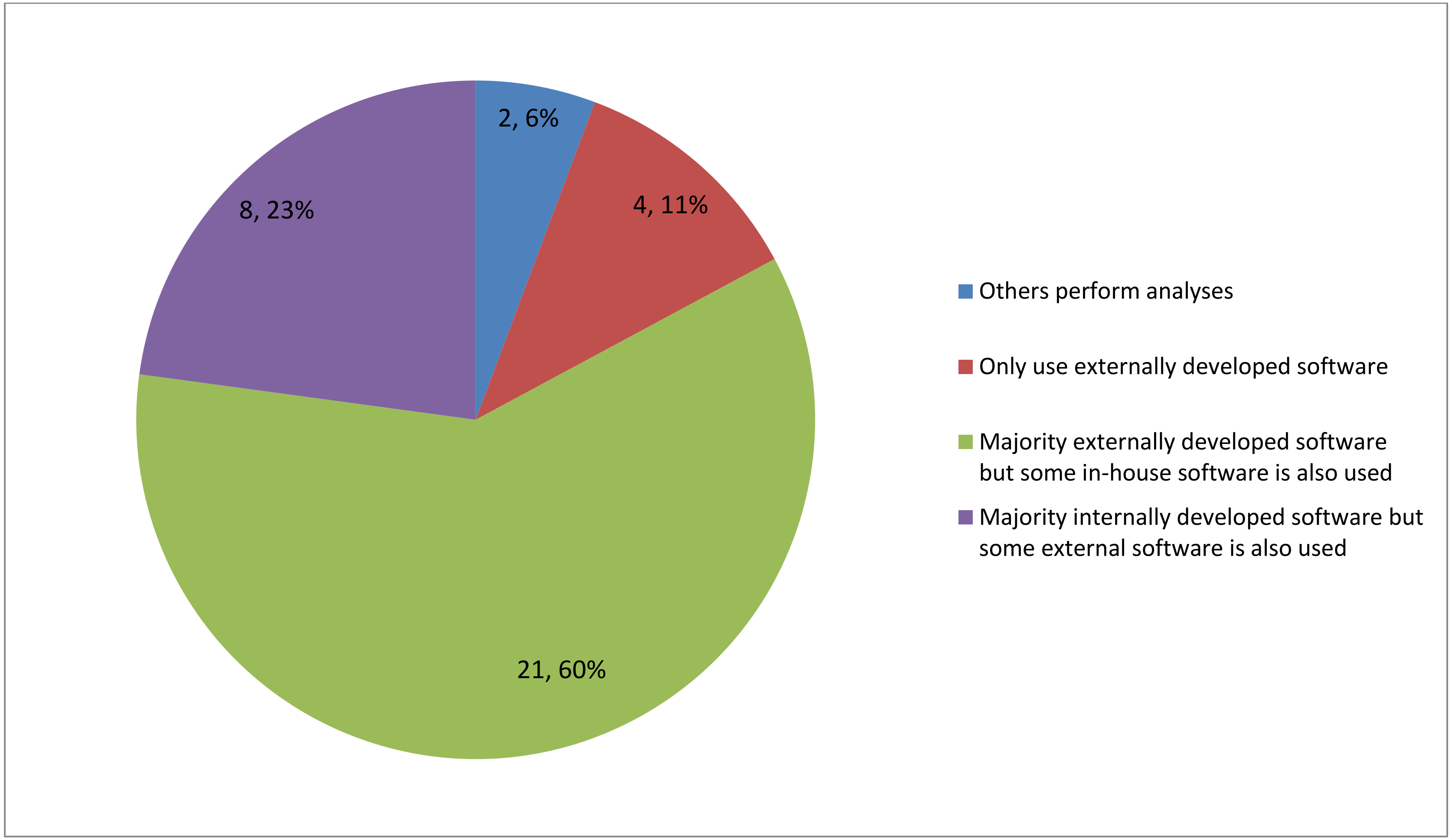

Supplement: Additional file 1: — Table S1. Costs of bacterial NGS by platform as reported by respondents at time of survey. Table S2. Volume of bacterial NGS performed annually by respondents. Table S3. Preferred number of strains per dispatch of PT for NGS. Table S4. Expected coverage while performing NGS for various taxa*. Figure S1. Organism and data transfer arrangements in place. Figure S2a. NGS capability of participating institutions. Figure S2b. Bioinformatics capability of participating institutions. Figure S3. Distribution of NGS access across technologies. Figure S4a. Distribution of top priority pathogens most commonly processed in participating institutions. Figure S4b. Distribution of pathogens most commonly processed in participating institutions. Figure S4c. Frequency of taxons genome-sequenced over passing year. Figure S5a. Main purpose of NGS experiments (mean scores). Figure S5b. Criteria for selecting pathogens for NGS experiments (mean scores). Figure S6. Library preparation methods employed for NGS. Figure S7. Perception of the importance of quality filtering during NGS analysis. Figure S8. Frequency of criteria used for QA/QC of assemblies. Figure S9. Use of bioinformatics software for analysis. [file 12879_2015_902_MOESM1_ESM.pdf]
